# Ovarian Cancer Mortality Predictors in Public Oncology Centers, Addis Ababa, Ethiopia: A Case–Control Study

**DOI:** 10.1002/cnr2.70537

**Published:** 2026-04-02

**Authors:** Abrham Tesfaye Habteyes, Jembere Tesfaye Deressa, Roza Teshome Kassa

**Affiliations:** ^1^ Department of Nursing College of Health Sciences, Ethiopian Defence University Bishoftu Ethiopia; ^2^ School of Nursing and Midwifery College of Health Sciences, Addis Ababa University Addis Ababa Ethiopia

**Keywords:** determinants, mortality, oncology centers, ovarian cancer

## Abstract

**Background:**

Ovarian cancer remains the most lethal gynecologic cancer and has the worst prognosis among all female reproductive malignancies worldwide. In Ethiopia, ovarian cancer is the third most prevalent malignancy among women, following breast and cervical cancers. Despite extensive research on the topic, evidence regarding the determinants of mortality among ovarian cancer patients remains limited. Therefore, the primary aim of this study was to identify predictors of ovarian cancer mortality among patients receiving care at oncology centers in Addis Ababa, Ethiopia.

**Methods:**

A facility‐based case–control study was conducted among women with ovarian cancer enrolled at oncology centers in Addis Ababa. A total of 137 cases and 274 controls were selected using the stratified sampling method. Data were extracted from patient records with a structured data extraction tool. The effect of each predictor variable on ovarian cancer mortality was estimated using binary logistic regression at the 5% level of significance and the final model's goodness of fit was tested with the likelihood ratio test.

**Results:**

This study included 411 ovarian cancer patients. Advanced FIGO stage (III&IV) (AOR: 2.85, 95% CI: [1.27–6.39]), advanced age (≥ 60) (AOR: 8.71; 95% CI: [3.89–19.48]), and comorbidity (AOR: 3.24, 95% CI: [1.69–6.19]) were found to be independent predictors of mortality, whereas urban residency (AOR: 0.36; 95% CI: [0.19–0.66]) and nonepithelial histological type (AOR: 0.12, 95% CI: [0.03–0.53]) were found to be protective factors. Moreover, patients not receiving pain medication had lower odds of mortality (AOR: 0.39; 95% CI: [0.22–0.69]).

**Conclusions:**

Multivariable logistic regression analysis identified the following factors as significant predictors of mortality among ovarian cancer patients: advanced FIGO stage, comorbidities, advanced age, nonepithelial histology, pain medication, and urban residency. Therefore, early detection through timely clinical evaluation and diagnosis and treatment should be emphasized. Moreover, patients identified with significant predictors of mortality should receive targeted clinical attention and closer follow‐up to improve survival outcomes.

**Abbreviations**
FIGO: International Federation of Gynecology and Obstetrics
GLOBOCAN: Global Cancer Statistics Database
HIV: human immunodeficiency virus
OC: ovarian cancer

## 1 Background

Ovarian cancer is a malignant tumor originating in the ovaries that can metastasize beyond the primary site [1]. It ranks as the 7th most frequently diagnosed malignancy worldwide and a major cause of cancer‐related death among women [2] and the third most common gynecological malignancy after cervical and uterine cancers [3]. In 2021, ovarian cancer accounted for approximately 299 000 new cases and 185 600 deaths globally, with an age‐standardized incidence rate of 6.7 per 100 000 women [4]. The global distribution of ovarian cancer cases varies across regions, with Asia accounting for the largest proportion of newly diagnosed cases (51.8%), followed by Europe (22.9%), North America (9.2%), Latin America (7.9%), Africa (7.4%), and Oceania (0.73%) [5]. Nevertheless, developing nations experience the greatest proportion of age‐specific mortality among ovarian cancer patients [6].

Ovarian cancer carries a poor prognosis, with global 5‐year survival rates of 45%–51%, largely due to late‐stage diagnosis [7]. Despite advances in early detection and treatment that have reduced mortality, effective screening remains limited [8]. Its insidious onset and nonspecific symptoms make early diagnosis challenging [8, 9], with approximately 75% of patients presenting with advanced‐stage disease at diagnosis [10]. This high mortality and late presentation have earned ovarian cancer the designation of a “silent killer” [8].

Regional distribution showing that Africa contributes a disproportionate share of ovarian cancer mortality relative to incidence, with Sub‐Saharan Africa contributing roughly 7%–8% of global cases and deaths [11]. Ovarian cancer deaths in Ethiopia increased substantially, rising from about 492 deaths (2000) to 1211 deaths (2021), a > 140% increase [12]. Survival remains substantially lower than in high‐income countries and varies across the region. A population‐based study across six African countries reported a 45% overall 5‐year survival [13], while Ethiopian hospital‐ and registry‐based cohorts from Addis Ababa show only ~34% 5‐year survival [14]. This is due to multifactorial contributing factors, including late‐stage presentation, delayed diagnosis, and limited access to timely, standard treatment [15].

Efforts to reduce mortality and improve quality of life for ovarian cancer patients remain critical priorities [16, 17], driven by rising incidence and mortality due to longer life expectancy, population growth, and increased risk associated with economic development [16]. Several studies have identified key predictors of ovarian cancer mortality, including advanced FIGO stage at diagnosis, residual tumor after cytoreductive surgery, older age, poor performance status, waiting time, treatment modality, and suboptimal therapy [18, 19, 20, 21]. Despite this, data on the demographics, clinical characteristics, and treatment patterns of ovarian cancer patients remain limited [22].

Although predictors of ovarian cancer mortality have been widely described in high‐income countries [23], their impact may differ substantially in low‐resource settings. In Ethiopia, oncology services are concentrated in a few tertiary hospitals in the capital city, which may lead to delayed diagnoses and limited access to care [21]. Patients in low‐ and middle‐income countries often experience poorer access to diagnostic and treatment services, later stage at presentation, and lower survival compared with those in high‐income countries [23]. Context‐specific evidence is therefore essential to understand how established mortality predictors operate within this constrained health system environment.

The Ethiopian government has prioritized the prevention and control of noncommunicable diseases, with a focus on reducing cancer incidence and mortality and improving patients' quality of life [24]. Despite these efforts, data on the determinants of ovarian cancer mortality in Ethiopia remain limited [15]. Multiple factors influence ovarian cancer mortality, and identifying and addressing these determinants could help reduce deaths, inform prevention strategies, and improve standards of care [25]. Moreover, it is essential to evaluate current treatment practices and to guide the future direction of cancer care in the country [22]. Therefore, this case–control study aimed to identify and quantify predictors of ovarian cancer mortality in public oncology centers in Addis Ababa, filling a critical gap in local and regional evidence.

## 2 Methods and Materials

### 2.1 Study Design, Area and Period

This study employed a facility‐based unmatched case–control design to identify predictors of mortality among women diagnosed with ovarian cancer at public oncology centers in Addis Ababa, Ethiopia. A case–control design was chosen due to the availability of patient records and limitations in follow‐up data. The study included Zewditu Memorial Hospital (ZMH), St. Paul's Hospital Millennium Medical College (SPHMMC), and Tikur Anbessa Specialized Hospital (TASH). SPHMMC and TASH are tertiary‐level public oncology centers that have been providing services for over 5 years, receiving referrals from primary and secondary healthcare facilities across the country. As access to specialized oncology services is centralized, patients attending these centers represent referrals from diverse geographic and socioeconomic backgrounds. Data collection for the study took place between December 5, 2024 and March 13, 2025.

### 2.2 Population

The source population included all women diagnosed with ovarian cancer at public oncology centers in Addis Ababa. The study population comprised the patients who were treated in the public oncology units and met the inclusion criteria.

- *Cases:* Women with histologically confirmed ovarian cancer who died during the study period (March 2020 to 2025).
- *Controls:* Women with ovarian cancer who remained alive at the end of follow‐up period.

### 2.3 The Eligibility Criteria

All patients with a new diagnosis of ovarian cancer received treatment at the selected public oncology units of the selected hospitals and who had complete medical records during the study period were eligible for inclusion. Patients were excluded if their medical records were incomplete or unavailable; of the total 784 records reviewed, 58 were excluded due to missing key clinical information required for analysis. Patients diagnosed at other health facilities and subsequently referred to the selected hospitals were also excluded to ensure consistency and completeness of baseline diagnostic and treatment data. Additionally, patients with a prior history of ovarian cancer treatment were excluded.

### 2.4 Sample Size Determination

The sample size was calculated using the double population proportion formula, based on key predictor variables identified in a pilot study conducted at TASH 15 days prior to data collection, which included 25 cases and 25 controls. Among these variables, comorbidity was selected as the primary predictor, as it yielded the largest required sample size. In the pilot study, 26% of controls and 44% of cases had preexisting comorbidities. Using a case‐to‐control ratio of 1:2, a 95% confidence level, and 80% power, the total sample size was determined to be 411 participants, comprising 137 cases and 274 controls.

### 2.5 Sampling Technique and Procedure

A stratified sampling procedure was used to select cases and controls separately. A total of 784 medical records of patients with a primary ovarian cancer diagnosis and newly initiated treatment at TASH (406), SPHMMC (341), and ZMH (37) between March 1, 2020, and 2025 were reviewed. Records meeting the inclusion criteria were identified by data collectors. Finally, 137 cases and 274 controls were selected using a computer‐generated lottery method after proportional allocation across the three hospitals.

### 2.6 Study Variables

#### 2.6.1 Dependent Variable

Mortality status of ovarian cancer patients.

#### 2.6.2 Independent Variables

In this study, 11 independent variables were comprised and analyzed, including sociodemographic and reproductive health factors (residential address, age at diagnosis, and parity), clinical factors (FIGO stage at diagnosis, histology type, comorbidity, and HIV status), and treatment‐related factors (type of treatment initiated, treatment duration, treatment completion, and use of pain medication).

### 2.7 Data Collection Tools and Procedure

Data were collected using a structured abstraction format developed from previous studies to capture risk factors for ovarian cancer mortality. The content of the variables was reviewed by senior experts, and the format was pretested before use. Medical records were selected based on eligibility criteria, and patient outcomes were obtained from the records. Data were collected by six trained oncology nurses and supervised by an experienced MSc midwife.

### 2.8 Data Quality Control

To ensure data quality, supervision was conducted throughout the data collection period, and appropriate data extraction tools were developed. Senior experts reviewed the content validity of the abstraction format. A pretest and pilot study involving 25 cases and 25 controls at TASH were conducted to assess the appropriateness of the data collection tools, refine the design, and finalize the sample size. Necessary amendments were made based on the results. All completed forms were checked for completeness, and data collectors and the supervisor received 1 day of training on the data collection procedures.

### 2.9 Data Processing and Analysis

Data were checked for completeness and consistency, then cleaned, coded, and entered into EpiData v3.1 before being exported to SPSS v27 for analysis. Descriptive statistics were used to summarize the study population. Due to incomplete follow‐up information, logistic regression was used to identify predictors of mortality, rather than time‐to‐event analysis. Therefore, mortality was analyzed as a binary outcome (dead vs. alive) using logistic regression. Odds ratios (ORs) were calculated to assess the strength of associations between independent variables and ovarian cancer mortality. Variables with *p* ≤ 0.25 in the bivariable model were included in the multivariable logistic regression model because bivariable tests may have low power to identify important predictors or confounders; a more permissive cutoff reduces the risk of excluding variables that become significant after adjustment [26, 27]. In the final model, six predictors were included with a total of 137 mortality events, resulting in approximately 22.8 events per variable, which exceeds the commonly recommended minimum of 10 events per variable and indicates low risk of overfitting. Multicollinearity among covariates was assessed using variance inflation factors (values between 1 and 5; tolerance > 0.2), and no evidence of significant collinearity was observed. Model fit was evaluated using the Hosmer–Lemeshow test, which indicated a good fit (*χ* ^2^ = 6.74, df = 8, *p* = 0.57). Statistical significance in the multivariate model was set at *p* < 0.05.

## 3 Results

### 3.1 Sociodemographic, Reproductive and Clinical Characteristics of the Study Participants

A total of 411 ovarian cancer patients who met the inclusion criteria were included in the final analysis. The mean age at diagnosis was 46.98 years (SD ± 16.15), the largest proportion of patients 161 (39.2%) were younger than 40 years of age, and 178 (43.3%) had parity of four or more. At diagnosis, 251 (61.1%) patients presented with advanced‐stage disease (FIGO stage III–IV). Epithelial tumors were the predominant histological type, accounting for 331 (80.5%) cases. Approximately one‐quarter of the patients (96, 23.4%) had comorbid medical conditions, and the majority (360, 87.6%) were HIV‐negative (Table 1).

**TABLE 1:** Sociodemographic, reproductive and clinical characteristics of ovarian cancer patients at public oncology centers, Addis Ababa, Ethiopia (*n* = 411).

Variables	Category	Status		Chi squared (*χ* ^2^)	*p*
		Case no. (%)	Control no. (%)		
Age	< 40	19 (13.9%)	142 (51.8%)	90.388	0.001
	40–49	19 (13.9%)	50 (18.3%)		
	50–59	26 (18.9%)	46 (16.8%)		
	≥ 60	73 (53.3%)	36 (13.1%)		
Residency	Rural	82 (59.9%)	119 (43.4%)	9.859	0.002
	Urban	55 (40.1%)	155 (56.6%)		
HIV status	Positive	14 (10.2%)	17 (6.2%)	3.627	0.163
	Negative	114 (83.2%)	246 (89.8%)		
	Unknown	9 (6.6%)	11 (4.0%)		
FIGO stage	Stage I and II	18 (13.1%)	142 (51.8%)	57.495	0.001
	Stage III and IV	119 (86.9%)	132 (48.2%)		
Comorbidity	Yes	53 (38.7%)	43 (15.7%)	26.972	0.001
	No	84 (61.3%)	231 (84.3%)		
Histology type	Epithelial	135 (98.5%)	196 (71.5%)	42.497	0.001
	Nonepithelial	2 (1.5%)	78 (28.5%)		
Number of parities	0–1	24 (17.5%)	104 (37.9%)	18.841	0.001
	2–3	38 (27.7%)	67 (24.5%)		
	≥ 4+	75 (54.8%)	103 (37.6%)		

### 3.2 Treatment Related Characteristics of the Study Participants

Chemotherapy and surgery were the predominant modes of treatment for 275 (66.9%) patients. More than half, 214 (52.1%), of the women received treatment for less than 18 months, 242 (58.9.4%) did not complete their treatment, and less than half, 153 (37.2%) of the women received pain medication (Table 2).

**TABLE 2:** Treatment related characteristics of ovarian cancer patients at public oncology centers, Addis Ababa, Ethiopia (*n* = 411).

Variables	Category	Status		Chi squared (*χ* ^2^)	*p*
		Case no. (%)	Control no. (%)		
Type of treatment	Chemo‐surgery	115 (83.9%)	160 (58.3%)	73.379	0.001
	Surgery	4 (2.9%)	107 (39.1%)		
	Chemotherapy	8 (5.8%)	6 (2.2%)		
	Palliative care	5 (3.7%)	0 (0.0%)		
	Others	5 (3.7%)	1 (0.4%)		
Duration of treatment	≤ 18 months	55 (40.1%)	159 (58.0%)	11.704	0.001
	> 18 months	82 (59.9%)	115 (41.9%)		
Treatment completed	Yes	30 (21.9%)	139 (50.7%)	31.359	0.001
	No	107 (78.1%)	135 (49.3%)		
Pain medication received	Yes	78 (56.9%)	75 (27.4%)	34.156	0.001
	No	59 (43.1%)	199 (72.6%)		

### 3.3 Determinants of Ovarian Cancer Patient Mortality

The association between the outcome and predictor variables was assessed using binary logistic regression analysis. Initially, bivariable analysis was conducted, and variables with a *p*‐value < 0.25 were considered candidates for inclusion in the multivariable logistic regression model. These variables included age at diagnosis, residential area, FIGO stage, histological type, preexisting comorbidity, parity, treatment completion status, pain medication use, and duration of treatment. Then, multivariable logistic regression analysis was performed by adjusting the effect of confounders using all variables with a *p*‐value of < 0.25 in the bivariate analysis and noncollinear explanatory variables. Finally, six variables were found to be strong predictors of ovarian cancer mortality in the multivariate logistic regression model (Table 3). Older women (> 60 years old) had an 8.71 times higher risk of death than did those under 40 years old (AOR: 8.71; 95% CI: [3.89–19.48]). Likewise, women with advanced clinical Stage (III and IV) disease at the time of ovarian cancer diagnosis were 2.85 times more likely to die than those with early FIGO clinical Stage (I and II) disease (AOR: 2.85, 95% CI: [1.27–6.39]).

**TABLE 3:** Bivariable and multivariable logistic regression analysis of ovarian cancer patients at public oncology centers, Addis Ababa, Ethiopia (*n* = 411).

Factors	Category	Bivariable COR (95% CI)	Multivariable AOR (95% CI)	*p*
Age at diagnosis	< 40	Ref.	Ref.	
	40–49	2.84 (1.39–5.79)	1.63 (0.71–3.72)	0.246
	50–59	4.22 (2.14–8.33)	2.63 (1.15–6.04)*	0.022
	≥ 60	15.16 (8.1–28.3)	8.71 (3.89–19.48)*	< 0.001
Residency	Rural	Ref.	Ref.	
	Urban	0.52 (0.34–0.78)	0.36 (0.19–0.66)*	< 0.001
Number of parities	0–1	Ref.	Ref.	
	2–3	2.46 (1.35–4.46)	1.46 (0.67–3.19)	0.338
	≥ 4+	3.16 (1.85–5.38)	0.97 (0.44–2.13)	0.937
FIGO stage at diagnosis	Early stage	Ref.	Ref.	
	Advanced stage	7.11 (4.11–12.32)	2.85 (1.27–6.39)*	0.011
Histologic type	Epithelial	Ref.	Ref.	
	Nonepithelial	0.04 (0.01–0.15)	0.12 (0.03–0.53)*	0.005
Comorbidity	No	Ref.	Ref.	
	Yes	3.39 (2.11–5.44)	3.24 (1.69–6.19)*	< 0.001
Treatment completion	No	Ref.	Ref.	
	Yes	0.29 (0.19–0.44)	0.77 (0.37–1.61)	0.489
Duration of treatment	≤ 18 months	Ref.	Ref.	
	> 18 months	2.06 (1.36–3.13)	1.15 (0.59–2.20)	0.683
Pain medication received	Yes	Ref.	Ref.	
	No	0.29 (0.19–0.44)	0.39 (0.22–0.69)*	0.001

*Note:* * indicates that the variables significantly associated with the outcome variable in multivariable analysis, *p*‐value: reflect the statistical significance of the association between each independent and outcome variable in the multivariable logistic regression model, Ref: indicates the baseline comparison group, Early stage: Stage I and II, Advanced stage: Stage III and IV.

Abbreviations: AOR, adjusted odds ratio; CI, confidence interval; COR, crude odds ratio.

The likelihood of mortality among ovarian cancer patients who lived in urban areas decreased by 64% compared to those who lived in rural areas (AOR: 0.36; 95% CI: [0.19–0.66]). Similarly, women with the nonepithelial histologic type of ovarian cancer had an 88% lower mortality rate than their counterparts (AOR: 0.12, 95% CI: [0.03–0.53]), and patients who did not receive pain medication had 61% lower odds of mortality (AOR: 0.39; 95% CI: [0.22–0.69]); however, this variable likely reflects underlying disease severity and palliative status rather than a protective effect. Furthermore, ovarian cancer patients who had comorbidities had a 3.24 times higher risk of death than did their counterparts (AOR: 3.24, 95% CI: [1.69–6.19]) (Table 3).

## 4 Discussion

Ovarian cancer remains among the fatal gynecologic cancers, exhibiting higher mortality rates than other female reproductive malignancies [28]. This case–control study aimed to identify the determinants of ovarian cancer mortality at public oncology centers in Addis Ababa, Ethiopia. While the predictors identified in this study are consistent with global literature, their impact in the Ethiopian context may be amplified by structural health system constraints. Oncology services are centralized in a limited number of tertiary hospitals, potentially contributing to delayed diagnosis, treatment interruptions, and disparities between urban and rural populations [21, 23, 29]. Therefore, this study provides context‐specific evidence to inform risk stratification, resource allocation, and targeted interventions within Ethiopia's evolving cancer care system.

Advanced age at diagnosis (≥ 60) was strongly associated with mortality. This finding is consistent with different studies conducted in various countries [10, 30]. This might be due in part to a higher burden of comorbidities in elderly women that complicate cancer therapy, lead to undertreatment or nonstandard treatment, and increase the risk of treatment‐related complications and death [31]. Moreover, this may be explained in part by age‐associated declines in immune function and other biological processes that reduce antitumor responses and impair therapeutic effectiveness in older patients [32, 33].

Advanced Stage (III and IV) at diagnosis was strongly associated with mortality. This finding is in agreement with several studies conducted in different developed and developing countries [14, 30, 34]. This could be because ovarian cancer symptoms are vague and nonspecific, resulting in a delayed diagnosis, and tumor biological characteristics, such as aggressiveness and resistance to treatment, can also contribute to a higher mortality rate in women with advanced stage disease [35, 36]. Additionally, this may be related to limited access to specialized oncology services and reduced treatment efficacy in patients with late‐stage disease.

Patients with comorbidities had higher mortality, consistent with previous studies [37]. This could be because comorbidities have an impact on a woman's overall health and immune function, making it more difficult for her body's defense mechanism to fight cancer and recover from therapy. This also increases the chance of life‐threatening complications during treatment [38, 39]. Moreover, the presence of comorbidities may mask or obscure ovarian cancer symptoms, resulting in delayed detection and treatment.

Patients with nonepithelial histologic types had lower mortality than those with epithelial types. This finding aligns with previous studies [40, 41], possibly due to the nature of the epithelial tumor, which is aggressive and heterogeneous, does not cause symptoms in the early stages, and has a higher likelihood of spreading to other parts of the body than other histologic types of ovarian cancer; this can result in a poorer prognosis and a higher mortality rate [42].

Urban residence was associated with lower mortality compared to rural residence. This finding is in agreement with a study [43]. The observed association between urban residence and lower mortality may reflect differences in healthcare access, earlier diagnosis, availability of specialized oncology services, and treatment adherence. However, residual confounding and selection bias cannot be entirely excluded. Cancer treatment facilities are concentrated in the country's capital city or urban areas; therefore, most cancer patients are referred to the central level, which could result in a delay in diagnosis and treatment and increase the death rate.

The current findings also noted that patients who did not receive pain medication had lower odds of mortality than women who had not taken pain medication. Patients requiring analgesics are more likely to have advanced‐stage disease or to be receiving palliative care, reflecting a higher symptom burden commonly observed in advanced ovarian cancer [44]. It is important to note that pain medication use should be interpreted as a marker of advanced disease or higher symptom burden rather than serving as an independent predictor of mortality.

This study is the first to identify determinants of mortality among patients with malignant ovarian tumors in Ethiopia. However, it has several limitations. First, data quality was affected by the reliance on handwritten and manually stored medical records. Due to incomplete follow‐up data, we were unable to conduct time‐to‐event survival analysis, and logistic regression was used instead. Second, information relating to socioeconomic and reproductive health characteristics such as occupation, educational level, age at menarche, unexplained infertility, age at first birth, and age at menopause was not documented on the chart and was not included in this study. Third, patients with incomplete medical records and those diagnosed at other facilities prior to referral were excluded from the study. These exclusions may have introduced selection bias, as excluded patients could differ systematically in disease severity, access to care, or socioeconomic status; this could potentially lead to underestimation or overestimation of the observed associations. Therefore, the findings should be interpreted with consideration of this potential limitation. Finally, this study was conducted in three public tertiary oncology centers in Addis Ababa. Although these hospitals serve as major national referral centers receiving patients from various regions of Ethiopia, women who were unable to access tertiary care services, particularly those from rural or socioeconomically disadvantaged areas, may not be represented. Therefore, selection bias cannot be excluded.

## 5 Conclusion

The multivariable logistic regression analysis identified advanced FIGO stage, comorbidities, and older age at diagnosis as significant predictors of mortality among ovarian cancer patients. In contrast, nonepithelial histologic type and urban residence were associated with lower odds of mortality. Pain medication use, likely reflecting advanced or palliative disease status, was also associated with increased mortality. These findings highlight the critical importance of timely clinical evaluation, early diagnosis, and prompt initiation of treatment, as advanced‐stage disease is strongly associated with poor survival outcomes. Particular attention should be given to patients residing in rural areas, those diagnosed with epithelial ovarian cancer, older patients, and individuals with comorbid conditions. Strengthening early detection strategies, improving equitable access to oncology services, and ensuring comprehensive management of comorbidities may help reduce ovarian cancer mortality in this setting.

## 6 Implications of the Study

The findings of this study have several important clinical and public health implications. This study also provides context‐specific evidence to inform clinical practice, health system planning, and resource allocation aimed at reducing ovarian cancer mortality in Ethiopia and similar low‐resource settings. The study underscores the importance of early clinical evaluation and timely diagnosis, as advanced stage at presentation was strongly associated with mortality. Strengthening symptom awareness, referral systems, and access to diagnostic services may help reduce late‐stage detection. Higher mortality among older patients and those with comorbidities highlights the need for comprehensive and multidisciplinary care that addresses both cancer treatment and coexisting medical conditions.

The observed urban–rural disparity suggests inequities in access to oncology services, emphasizing the importance of improving access for rural populations, such as decentralization of oncology services, improved referral networks, and patient navigation support may help reduce these disparities. The association between epithelial histology and increased mortality supports the need for accurate and timely histopathologic diagnosis to guide treatment. Finally, pain medication use likely reflects advanced disease status, reinforcing the importance of early‐stage diagnosis and timely treatment initiation before progression to advanced or palliative stages.

## Author Contributions

A.T.H. conceived and designed the study, collected and analyzed the data, and drafted the manuscript. J.T.D. and R.T.K. assisted in data collection, interpretation of results, and critically revised the manuscript. All authors (A.T.H., J.T.D., and R.T.K.) read and approved the final version of the manuscript.

## Funding

The authors have nothing to report.

## Disclosure

Patients and members of the public did not participate in the design, execution, reporting, or dissemination of this study.

## Ethics Statement

Ethical approval was obtained from the Institutional Review Board of St. Paul's Hospital Millennium Medical College (IRB protocol: PM34/1017), where the study was implemented and data collection was conducted. The research protocol was reviewed at the departmental ethical committee level at Addis Ababa University for academic and scientific purposes; however, formal ethical clearance was granted by the IRB of St. Paul's Hospital Millennium Medical College in accordance with national research ethics requirements. The IRB approval authorized data collection at all participating public oncology centers in Addis Ababa. Permission letters were additionally obtained from each participating hospital to access medical records, in compliance with institutional and national regulations governing research involving patient data. The study was conducted in accordance with national ethical guidelines for medical and health research involving human participants and adhered to the ethical principles of the Declaration of Helsinki (2013 revision).

## Consent

As this study involved retrospective review of medical records with no direct patient contact and posed minimal risk to participants, the requirement for informed consent was waived by the IRB in accordance with the Ethiopian National Research Ethics Review Guideline (5th Edition) and applicable institutional regulations permitting waiver of consent for minimal‐risk research. In this case‐cohort study, no patient identifiers were used, and the data were anonymized. To maintain confidentiality, names and other personal identifiers were not included in the data collection tool.

## Conflicts of Interest

The authors declare no conflicts of interest.

## Data Availability

The data that support the findings of this study are available from the corresponding author upon reasonable request.
